# A Non-surgical Solution to Lacrimal Duct Obstruction After Laser-Assisted Subepithelial Keratectomy (LASEK) Surgery: A Case Report

**DOI:** 10.7759/cureus.97563

**Published:** 2025-11-23

**Authors:** Paarth Patel, Arshia Hamzehpour Savojbalaghi, Ambreen Wajid, Mikhail Volokitin

**Affiliations:** 1 Medicine, Touro College of Osteopathic Medicine, New York, USA; 2 Osteopathic Manipulative Medicine, Touro College of Osteopathic Medicine, New York, USA

**Keywords:** dacryocystorhinostomy, lacrimal duct obstruction, lasek, ophthalmology, osteopathic manipulative medicine

## Abstract

This report discusses a 24-year-old patient who experienced persistent lacrimal duct obstruction and excessive tearing following a successful bilateral laser-assisted subepithelial keratectomy procedure. Despite the use of conventional treatments, including steroid therapy, punctal dilation, and antibiotics, the issue persisted. The patient ultimately benefited from osteopathic manipulative medicine techniques, achieving significant improvement without the need for dacryocystorhinostomy surgery. This case highlights an innovative, non-invasive approach to a rare postoperative complication.

## Introduction

Laser-assisted subepithelial keratectomy (LASEK) is a well-established procedure for correcting refractive errors [1]. Although direct injury to the lacrimal system during LASEK is anatomically unlikely, postoperative inflammation, alterations in tear film dynamics, and topical medication use may contribute to secondary lacrimal drainage dysfunction in susceptible individuals [2]. Such cases can lead to nasolacrimal duct obstruction, resulting in persistent epiphora and discomfort. The standard treatment for unresolved lacrimal obstruction often involves surgical intervention such as dacryocystorhinostomy (DCR) [3]. However, osteopathic manipulative medicine (OMM) offers a physiologic, non-surgical approach aimed at improving lymphatic and venous drainage, reducing fascial strain, and restoring functional balance within the craniofacial region [4,5]. This case report presents the successful resolution of post-LASEK lacrimal obstruction using targeted OMM techniques.

## Case presentation

**Figure 1:**
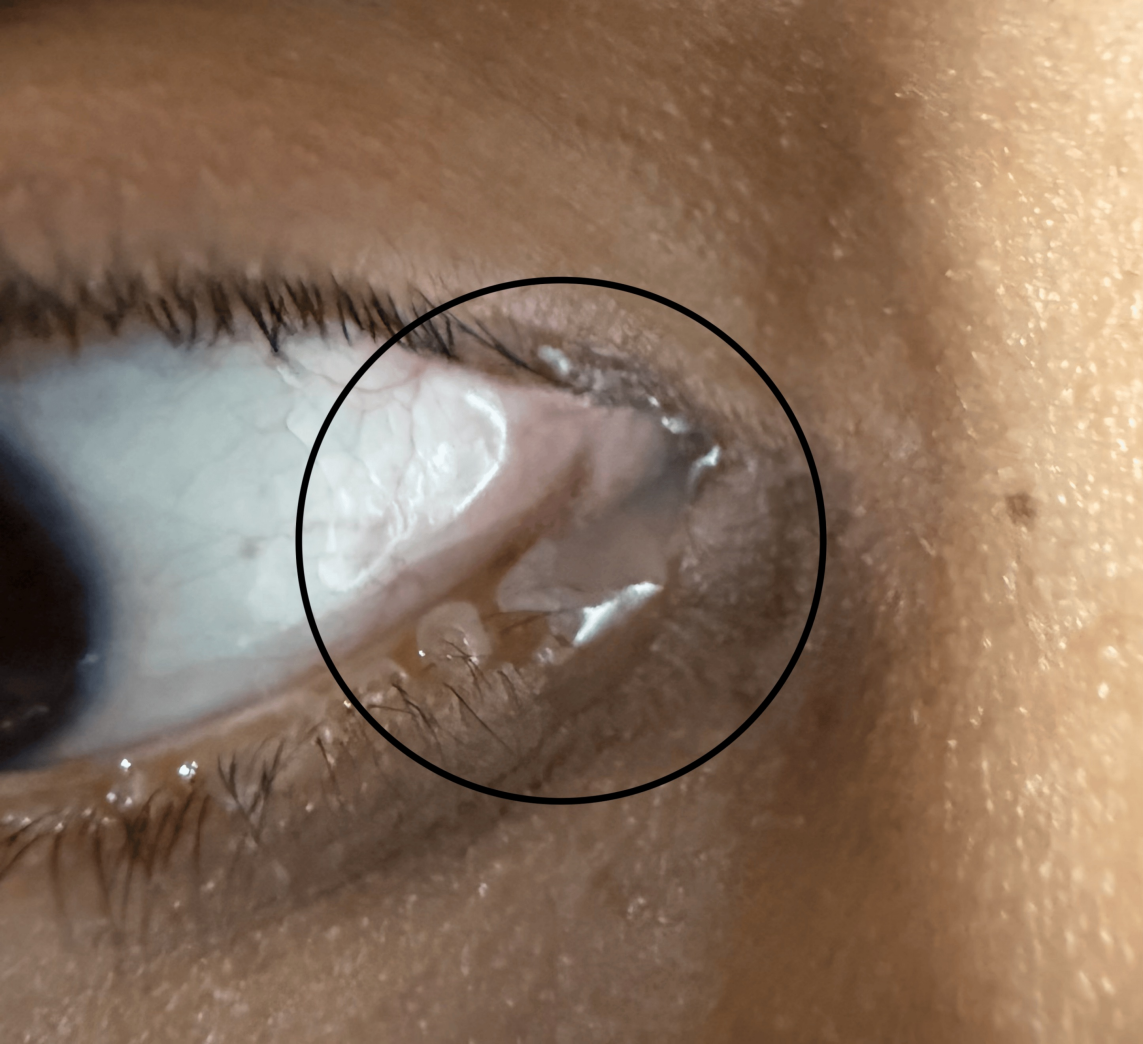
Epiphora of the right eye before punctual dilation

A 24-year-old patient with no significant medical history underwent bilateral LASEK in July 2024 to eliminate the need for corrective eyewear. Full re-epithelialization of the cornea was noted at six days postoperative; however, a sterile infiltrate appeared bilaterally. The patient was prescribed prednisolone acetate four times daily to reduce sterile infiltrate and prevent regression. Additionally, the patient received bilateral lower puncta cautery to decrease dry eye symptoms. One month later, the patient presented with symptoms of epiphora in the right eye, where excessive tearing was noted (Figure 1).

**Figure 2:**
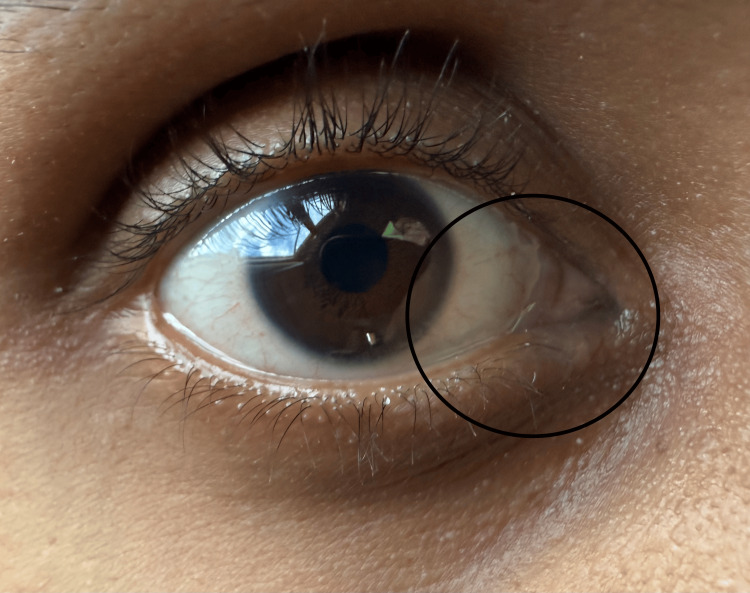
Epiphora of the right eye following punctual dilation and irrigation

Punctal dilation and irrigation were performed to relieve symptoms, but no changes were noted, as observed in Figure 2. The patient was referred for an oculoplastics evaluation, where 100% reflux was noted during the lacrimal duct irrigation test. DCR was recommended as the next line of treatment [3]. An otorhinolaryngology (ENT) consultation, along with a CT scan without contrast, revealed no significant findings or structural abnormalities. As a result, the patient was cleared for DCR surgery, which was recommended as the best course of treatment for the presenting symptoms (Figures 1, 2). However, as an osteopathic medical student, the patient sought OMM from a faculty member at his institution. The osteopathic physician recommended the patient perform a nasal suture gaping technique and a lymphatic drainage effleurage [5,6]. The technique consists of applying steady but slight posterolateral pressure on the glabella of the frontal bone and gentle disengagement of the nasal bones from the frontal [6]. The patient performed these techniques multiple times a day for approximately four weeks, resulting in significant improvement upon re-evaluation.

Postoperative recovery

The patient’s recovery initially followed a typical course, with full re-epithelialization by postoperative day six. Mild sterile infiltrates were identified and managed four times daily with prednisolone acetate. However, persistent epiphora did not resolve despite standard treatments, including punctal dilation, lacrimal duct irrigation, antibiotics, and steroid tapering [2,3].

Challenges and findings

**Figure 3:**
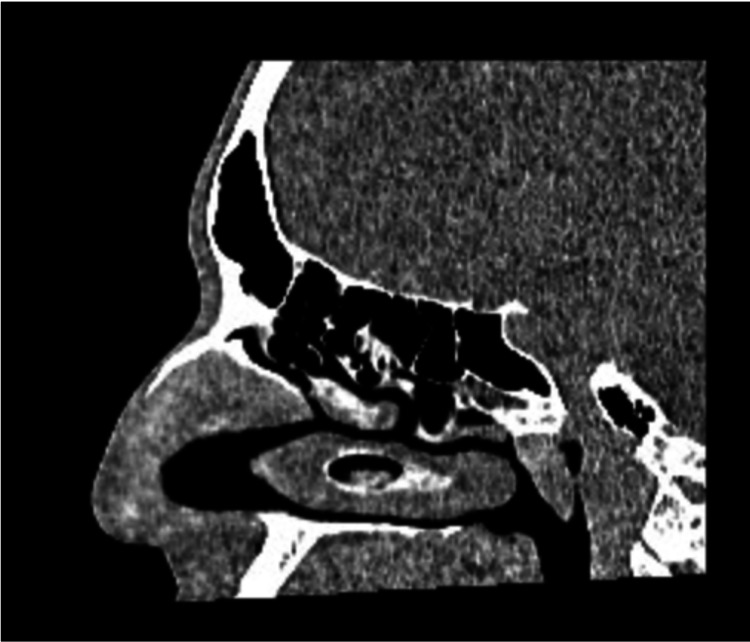
Normal anatomy revealed on CT scan of the affected area CT, computed tomography.

**Figure 4:**
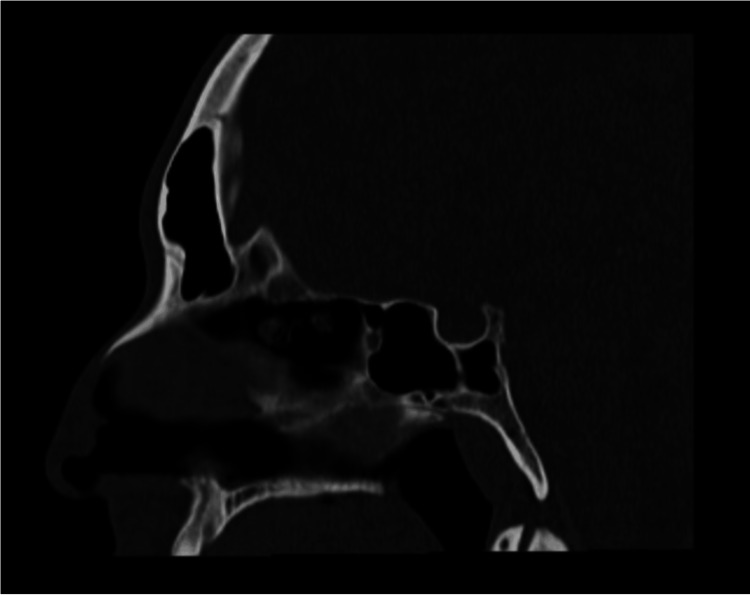
CT scan showing the absence of abnormalities and tumors in the nasolacrimal system. CT, computed tomography.

Persistent obstruction of the nasolacrimal duct was confirmed through irrigation tests, which demonstrated complete fluid reflux. A CT scan (Figure 3) revealed normal nasal anatomy, ruling out structural abnormalities affecting lacrimal drainage. Based on these findings, the ENT and oculoplastics teams recommended DCR surgery as the definitive treatment [3]. The CT scan (Figure 4) ordered by the ENT team specifically aimed to assess any nasal abnormalities that could be contributing to the obstruction.

Intervention

The patient, a medical student, sought advice from an osteopathic faculty member. Following the guidance provided, the patient began a regimen of nasal suture gaping exercises and lymphatic drainage techniques, which were repeated multiple times a day [5,6]. The student was advised to complete the exercises for two to three minutes per session, three to four times daily, over the course of approximately one month. The nasal suture gapping technique involved spreading the frontal bone from the nasal bone, restoring the normal motion of the area, and improving the symptoms of congestion, facial pain, sinusitis, and nasal obstruction [7]. The decompressive nature of the technique allows the flow of passages nearby, such as the lacrimal duct and sinuses [7]. The lymphatic drainage technique of the nasal duct helps move the tears down its physiologic path and restores the ability of the lacrimal sac to drain into the inferior meatus of the nasal cavity [8].

Outcome

Within four weeks of starting the OMM regimen, the patient experienced a marked reduction in symptoms, including resolution of lacrimal sac swelling and improved tear drainage. There was a significant decrease in the number of episodes and a decrease in the amount of fluid trapped in the lacrimal apparatus since most of it was draining into the inferior meatus. Functionality of the nasolacrimal duct was restored, as evidenced by the patient’s ability to taste topical steroids in the nasopharynx, signaling duct patency [2]. As a result, the patient was able to avoid the previously planned DCR surgery [3].

## Discussion

**Figure 5:**
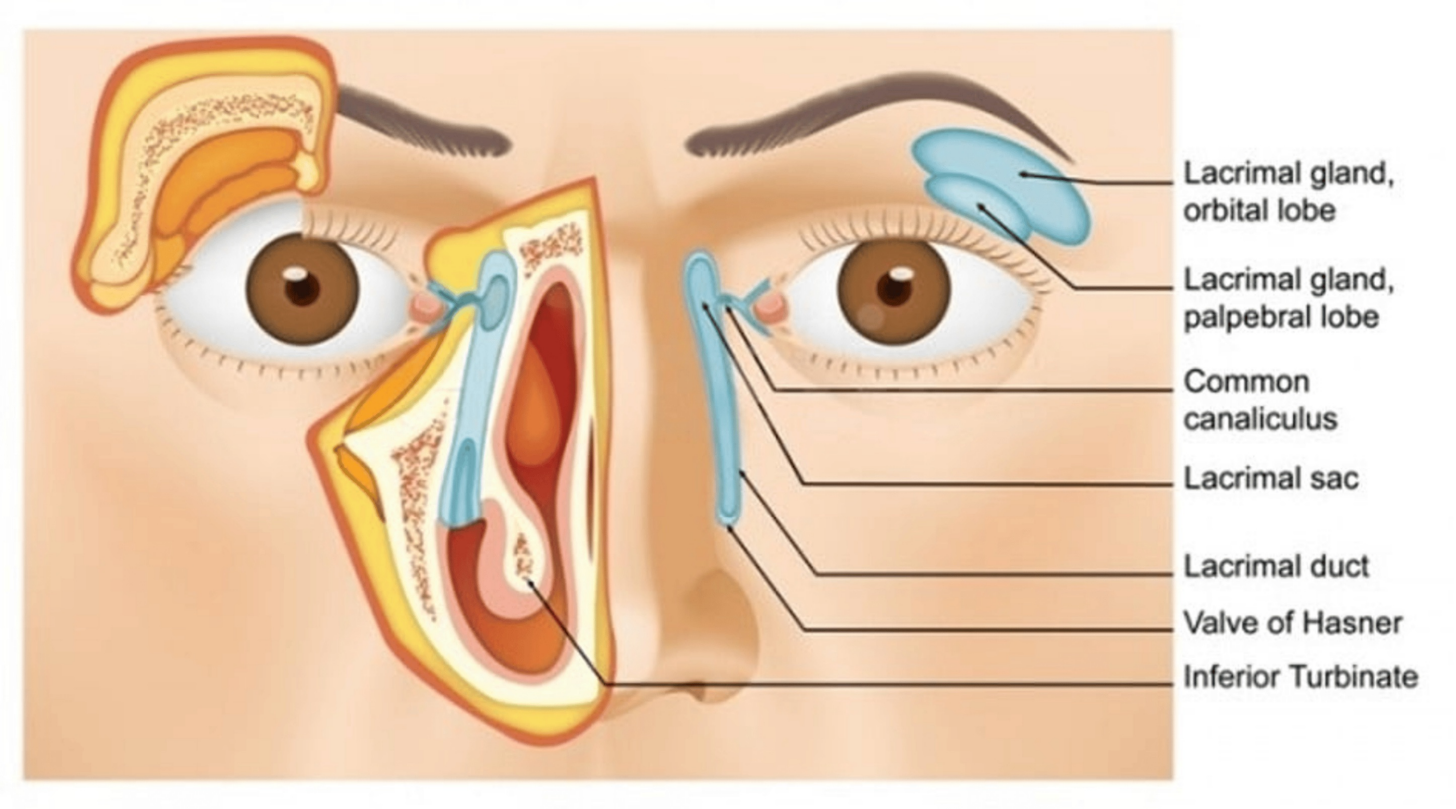
The normal anatomy of the nasolacrimal duct system [9]

Figure 5shows the normal anatomy of the nasolacrimal duct system, involving the drainage of tears from the lacrimal sac through the upper and lower puncta into the lacrimal duct, eventually exiting through the inferior meatus of the nose. Obstruction at any point along this passage can lead to impaired tear drainage, swelling of the lacrimal sac, and potential infection [8]. 

Patients who undergo LASIK or LASEK surgery sometimes experience tear duct plug insertion to slow tear drainage and alleviate dry eye symptoms. However, these plugs may not always remain in place. Alternatively, punctal cautery can offer a more effective method for slowing tear drainage [10,11]. When the patient presented with symptoms, both upper and lower puncta were noted to have returned to their normal size, but the obstruction was likely located further down the nasolacrimal system [2].

This case demonstrates the potential effectiveness of OMM techniques in resolving lacrimal duct obstruction unresponsive to conventional treatments. As the CT scan showed no anatomical abnormalities, the obstruction appeared functional. Nasal suturing and lymphatic drainage techniques proved crucial in restoring duct function. The non-invasive nature of this approach offers a valuable alternative for patients hesitant to undergo surgery.

This novel application of OMM warrants further investigation through larger studies to evaluate its broader applicability in managing similar cases of lacrimal system dysfunction [5,6,10].

## Conclusions

The use of OMM techniques represents a promising alternative for patients with lacrimal duct obstruction who wish to avoid surgical intervention. This case highlights the importance of exploring integrative and innovative solutions in postoperative care.
